# Knowledge, Attitudes, and Behaviors Regarding Lyme Borreliosis Prevention in the Endemic Area of Northeastern Poland

**DOI:** 10.3390/vaccines10122163

**Published:** 2022-12-16

**Authors:** Marta Wozinska, Kacper Toczylowski, Dawid Lewandowski, Ewa Bojkiewicz, Artur Sulik

**Affiliations:** Department of Pediatric Infectious Diseases, Medical University of Bialystok, Waszyngtona 17, 15-274 Bialystok, Poland

**Keywords:** *Borrelia burgdorferi*, Lyme borreliosis, tick bite, tick-borne diseases, Lyme vaccine

## Abstract

(1) Background: The incidence of Lyme borreliosis (LB) is increasing in Europe. The new LB vaccine is still in clinical development, thus the dissemination of knowledge about the disease is essential. We assessed the knowledge, attitudes and preventive practices (KAP) against tick-borne diseases (TBDs) of people living in the endemic area in northeastern Poland. (2) Methods: We surveyed 406 adults using a 37-item anonymous paper survey. The data were analyzed with regression models. (3) Results: The two most popular knowledge sources were the Internet and doctors, selected by 77.8% and 53.4%, respectively. Respondents felt moderately knowledgeable about TBDs and tick bite prophylaxis (median scores 5/10, and 6/10, respectively), considered TBDs to be a significant health threat (median 8/10), attributed high risk to tick mouthparts remaining in the skin after tick removal (median 10/10), and shared multiple misconceptions regarding LB transmission, symptoms, and management. General knowledge scores (GKS) about TBDs and tick protection practices scores (TPS) were moderate (65.0%; IQR, 55.8–71.7%, 63.6%; 54.5–72.7%, respectively). Only 48.0% had a positive attitude towards TBE vaccination. A recent tick-bite was associated with higher GKS (OR, 2.55; 95% CI, 1.27–5.10; *p* = 0.008), higher TPS (OR 4.76, 95% CI, 2.0–11.1; *p* < 0.001), and a positive attitude towards TBE vaccine (OR 2.10, 1.07–4.10, *p* = 0.030). A positive vaccine attitude was also associated with obtaining TBD knowledge from doctors and other verified sources (OR, 2.654, 1.66–4.23; *p* < 0.001). Age, place of residence, and frequent exposure to ticks in green areas were not associated with GKS, TPS, nor vaccine attitude. (4) Conclusions: Increased risk perceptions are associated with adoption of behaviors preventing TBDs. Medical professionals play an important role in communicating knowledge about TBDs. There is a need to revise current communication strategies with respect to tick bites and prevention of LB and other TBDs.

## 1. Introduction

Tick-borne diseases (TBDs) pose a great health risk to people living in endemic areas. The most common TBDs include Lyme borreliosis (LB) and tick-borne encephalitis (TBE) [1]. In Europe, the main vector of LB is the tick *Ixodes ricinus*, and the most cases are reported in the northern and central-eastern part of the continent [2,3].

LB is caused by *Borrelia burgdorferi* spirochetes of the *Spirochaetaceae* family, a part of the *Borrelia burgdorferi* sensu lato complex [4]. The *Borrelia burgdorferi* sensu lato complex is a very diverse group of bacteria. Based on various studies, numerous spirochete genospecies have been identified, and more are expected to be discovered in the future [4]. *Borrelia burgdorferi* sensu stricto, *Borrelia afzelli* and *Borrelia garinii* dominate among the species commonly infecting humans [5]. The heterogeneity of *Borrelia* spirochetes is one of the reasons for the diversity of clinical symptoms of the disease [6] that can affect skin, joints, heart, or the peripheral or central nervous system [7]. The serological diagnosis of LB is a great challenge due to the number of *Borrelia* species, their different geographical distribution, similarity to other pathogenic spirochetes resulting in the possibility of cross-reactions, and the location of the infection in immunologically privileged sites [8,9,10,11].

The incidence of LB is increasing in many countries. One of the reasons is the observed warming of the climate, which is conducive to the expansion of ticks to higher altitudes and towards poles. There is, however, an observable decrease in tick populations in geographical regions that became too hot and dry due to the climate change [12]. Additionally, the increase in LB incidence may be caused by the development of tourism, lifestyle changes and popularization of outdoor sports as well as better knowledge and notification of the cases by the physicians [1,13]. A similar increasing trend has been observed in Poland in recent decades [14]. Only in the period from 2020 to 2021 there was a decrease in the number of reported cases of LB, which could be related to the outbreak of the SARS-CoV-2 pandemic and the resulting limited access to diagnostics. This decrease in reported cases resulted from the effects of the pandemic on TBD surveillance rather than an actual decrease in the numbers of tickborne diseases. The data showed that incidence LB reduced compared to prior years [15]. In Poland, the highest incidence is recorded in the northeastern part of the country, where our research was conducted [14].

With increasing rates of LB all around the globe, as well as the challenging serodiagnostics and treatment, protecting people from the disease becomes more important than ever. The undoubted success of the TBE vaccine in reducing the incidence of tick-borne encephalitis in endemic areas indicates that it is the right way to reduce the incidence of LB [16]. Researchers have been searching for an effective vaccine against LB since the 1970s. A vaccine based on the outer surface protein A (OspA) lipoprotein was approved by the US Food and Drug Administration (FDA) in the United States in 1998 [17]. A number of unfavorable reports and the activity of anti-vaccine movements contributed to the withdrawal of this vaccine [18,19]. Recently, a new attempt to develop a vaccine has been made, for instance vaccine VLA15 which also targets the OspA of *Borrelia burgdorferi* [20,21] or LB vaccine using mRNA to encode tick saliva proteins [22].

Final registration and public acceptance of vaccination against *Borrelia* will provide real protection against the disease caused by the bacteria. Until then, tick bite prophylaxis and its consistent application is the only method of protection against the disease [23]. Avoiding places with high grass, hiking in the middle of the trails, avoiding leaf litter, wearing long-sleeved clothing, carrying out tick control on the whole body and removing ticks promptly is included to taking steps to protect from getting a tick bite. Research shows, that the use of repellent is one of the least frequently chosen methods of tick protection, probably due to the high cost and the false perception that these products may be toxic to the skin [24].

Assessment of knowledge, attitudes and practices (KAP) in the exposed population is an important element in the prevention of infection with tick-borne pathogens [25,26]. Previous studies pointed that TBD awareness among healthcare professionals [25,27], people living in endemic area [26], and foresters [28] was inadequate. However, respondents had a favorable attitude towards a future LB vaccine [25,28,29]. Increasing the knowledge through appropriate educational activities could improve TBD awareness, reduce the exposure to tick bites, and thus have a positive impact on public health. 

Although exposure to TBDs is particularly high in our geographic region, little is known about TBD awareness. The aim of our study is to assess the knowledge and attitudes of respondents to TBDs, in particular LB, as well as to learn about behaviors related to tick bites. 

## 2. Materials and Methods

### 2.1. Study Design and Setting

The survey, based on an anonymous questionnaire consisting of 37 questions, was conducted at the Department of Pediatric Infectious Diseases, Medical University of Bialystok in 2019–2021. The participants of the study were recruited among parents of children hospitalized in the above-mentioned department. All respondents lived in northeastern Poland, which is endemic for TBDs. The research was conducted with the consent of the Bioethics Committee of the Medical University of Bialystok (approval number R-I-002/489/2019)

The questionnaire assessed knowledge, attitudes, and preventive practices against TBDs. In questions assessing knowledge, we used multiple choice questions. Attitudes and practices were determined using a fully labelled 10-point Likert scale, ranging from “of little importance” to “highly important”.

In the area of knowledge, we asked respondents to choose TBDs from a list of various health conditions, identify a tick on an illustration, select correct tick bite management, and also point symptoms, ways of transmission and methods of treatment of LB. The General Knowledge Score (GKS) was calculated on the basis of the number of correct answers. To assess the attitude towards TBDs we asked respondents to compare disease burden that they associate with TBDs, cancer and cardiovascular disease (CVD). Additionally, the respondents were asked if tick mouthparts remaining in the skin after tick removal pose any health danger, and if they or their child received a postexposure prophylaxis with antibiotics after a tick bite. Preventive practices were assessed by determining preferable tick bite protection methods, and use of repellents. The responses were then rated, and the tick protection score (TPS) was calculated by adding +1 point to a sum for every proper method labelled by the respondent as important (7 points or more in the Likert scale) and for every ineffective method or unimportant repellent characteristic labelled as nonimportant (4 points or less). For instance, an individual receives 8 points if they reported buying a repellent in the last year, and gives at least 7 out of 10 points in the Likert’s scale (interpreted as important) for post exposure body inspection, washing clothes post exposure, using proper clothing while outdoors, treating clothes with repellents and applying topical repellents on skin, while gives 4 points or less (interpreted as “unimportant”) to use of electronic repellants and oral supplements. For giving 4 points or less to pleasant smell, natural ingredients and lack of DEET as qualities of a repellent, an additional 3 points were added to a maximum score of 11. Additionally, we calculated a second index of tick protection, named “poor repellent practice”, defined as answering two or more of the following: not buying repellents, preferring products without DEET, preferring products containing natural ingredients, and preferring products having a pleasant smell (defined as rating 5 points or more in the 10-point Likert’s scale). We also assessed attitudes to the tick-borne encephalitis vaccine, which is the only vaccine used in prevention of TBDs in humans currently.

### 2.2. Information about the Interviewees

The collected data included: demographic data (gender, age, education level, place of residence), frequency of staying in green areas, past and last year tick bites, history of tick bites in the respondents’ children. Additionally, the participants of the study were asked about the sources of knowledge about TBDs and the prevention of tick bites, as well as about the occurrence of TBDs in the immediate family.

### 2.3. Data Analysis

The quality of this survey was asserted by two small (n = 20) pilot phases, during which we distributed the first versions of the survey among the hospital staff. After collecting the feedback, we improved the quality of the survey and prepared the final version of the questionnaire. Data were collected with anonymous questionnaires printed on paper. Subsequently, it was entered and compared by 3 independent people using Microsoft Excel, which reduced the risk of errors. Vague responses were reviewed by the principal investigator to determine the correct answer. We removed surveys that were illegible or in which the majority of answers were missing. In the case of only a few key answers missing we included this questionnaire but accounted for missing data in the analysis. Before the analysis we checked if the proportion of missing data is below 5% in each question.

The multivariate binominal logit regression model with the backward stepwise method was used to calculate factors associated with knowledge about TBDs, and tick protection practices. The results are presented as odd ratios (OR) with corresponding 95% confidence intervals (95% CI). Variables describing all personal factors (age, sex, place of residence, education, exposure to tick bites) were included in these analyzes as confounders. The relative disease burden assessed by the respondents was compared in one-way mixed-effects ANOVA and Tukey’s multiple comparisons post hoc test. A *p*-value less than 0.05 was considered statistically significant. All analyses were performed using TIBCO Software Inc. (2017) Statistica, version 13 (Palo Alto, CA, USA) and GraphPad Prism version 9.4.0 for Windows, GraphPad Software (San Diego, CA, USA).

## 3. Results

### 3.1. Characteristics of the Study Group

After removing 59 incomplete surveys, we included 406 questionnaires collected from 325 women (82%, 325/397), 72 men (18%, 72/397), and 9 people (2%) who did not specify their gender. One hundred sixty-one respondents (41%) had been bitten by a tick in the past at least once, and 66 (16%) of them were bitten within the last year. Moreover, 21 (5%) respondents had been bitten by a tick more than once. Additionally, 123 (31%) respondents answered that their child had been bitten by a tick at least once, and 40 (10%) more than once (Table 1).

### 3.2. Knowledge

The median GKS result was 65.0% (interquartile range (IQR), 55.8%, 71.7%). Using the regression model, we analyzed the differences between the respondents with high level of knowledge (GKS score above the median value) and the respondents with low levels of knowledge (GKS below the median value) in order to identify variables correlated with the knowledge levels. Respondents with high levels of general knowledge more often graduated from a university (OR, 1.70, 95% CI, 1.01–2.85; *p* = 0.047), felt more confident about TBD and prophylaxis (OR, 2.62; 95% CI, 1.55–4.41, *p* < 0.001), were more likely to be bitten by a tick recently (OR, 2.55; 95% CI, 1.27–5.10, *p* = 0.008), and were less likely to have a poor repellent practice(OR, 0.58; 95% CI, 0.36–0.94). High level of knowledge was also associated with not accepting unverified treatment for Lyme borreliosis, for instance: supplementation with vitamins, homeopathy, hydrocolonotherapy, hyperbaric chamber therapy, ozone therapy, bioresonance therapy, chelation therapy and therapy with intravenous vitamin C. Unverified treatment for Lyme disease was more often suggested by respondents with lower GKS scores (OR, 1.75; 95% CI, 1.04–2.95, *p* = 0.036). Knowledge scores were not associated with sex, age, place of residence, and exposure to ticks in green areas. 

The majority of the respondents (77.8%) use the Internet as their source of information on TBDs and tick bite prophylaxis. The second most popular knowledge source were doctors selected by 53.4%. A total of 153 (37.7%) respondents relied on non-verified sources of knowledge about TBDs only (the Internet, friends, popular science literature, other people diagnosed with TBDs, TV and radio). Respondents who relied on verified sources of knowledge (doctors, pharmacists, official medical websites and textbooks) were more likely to be under 30 years of age (OR, 2.36, 1.21–4.61, *p* = 0.012), have a positive attitude towards TBE vaccines (OR 2.82, 1.71–4.64, *p* < 0.001), have a child bitten by a tick (OR 2.12, 1.19–3.77, *p* = 0.011), and were bitten by a tick anytime in the past (OR 2.50, 1.46–4.28, *p* = 0.001).

**Figure 1:**
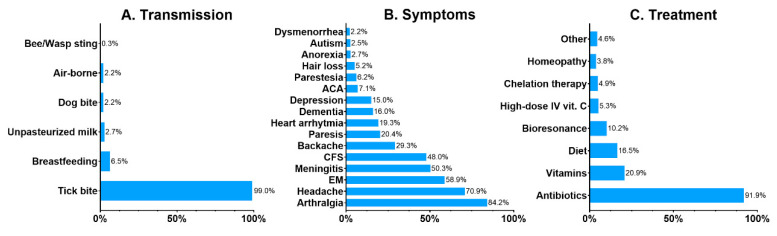
Respondents’ beliefs about the transmission (**A**), symptoms (**B**) and treatment (**C**) of Lyme borreliosis. Data presented as percentages. Abbreviations: ACA, acrodermatitis chronica atrophicans; CFS, chronic fatigue syndrome; EM, erythema migrans; IV, intravenous.

Common misconceptions and myths about the diseases and the principles of their prevention were revealed by the survey. Lyme disease, tick-borne encephalitis, and babesiosis were the best-known TBDs, selected by 96.8%, 83.5%, and 26.1% of the respondents. The least known TBDs were anaplasmosis (12.6%), tularemia (8.4%), rickettsiosis (7.1%) and ehrlichiosis (5.9%). Other health conditions were commonly attributed to tick bites: congenital LB was selected by 20.0%, autoimmune disorders by 9.1%, miscarriage by 4.9%, toxoplasmosis by 4.7%, and malaria by 2.7%. Only 77% of the respondents properly identified a tick on a picture. A bed bug (*Cimex lectularius*) and a dust mite (*Dermatophagoides pteronyssinus*) were mistaken for ticks by 10.6% and 5.9% of the respondents, respectively. An orb-weaver spider was selected by 2.6%, and a louse by 2.8%. Only 38.9% of the respondents were convinced that LB is curable, while 39.4% claimed that it is not, and 21.6% responded “I do not know”. Although the majority of the respondents (87.5%) know that LB is transmitted by tick bites, 12.5% are convinced that other means of transmission are possible, such as breastfeeding or drinking unpasteurized milk (Figure 1A). The majority of the respondents properly indicated common symptoms of LB such as arthritis (84.2%), headaches (70.9%), erythema migrans (58.9%), and nerve paresis (20.4%), but there were answers such as autism (2.5%) and hair loss (5.2%) (Figure 1B). A vast majority of the respondents (91.9%) correctly responded that antibiotics are the treatment for LB. However, 40.6% of the respondents selected alternative treatment for LB as well (Figure 1C).

### 3.3. Attitude

**Figure 2:**
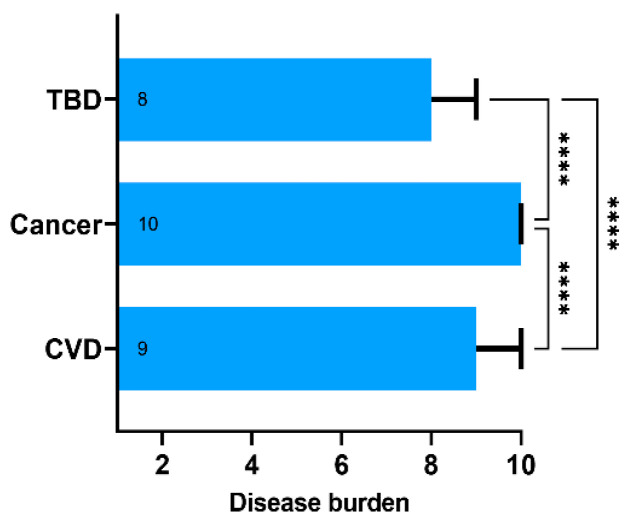
The subjective disease burden assessed using the 10-point Likert scale ranging from 1—“does not affect the human health” to 10—“poses significant public health burden”. Data presented as medians (boxes) and interquartile range (whiskers). The annotations within the bars represent the median values. **** represent *p*-value below 0.001 calculated with the one-way ANOVA followed by Tukey’s multiple comparisons test. Abbreviations: TBD, tick-borne diseases; CVD, cardiovascular diseases.

Using the 10-point Likert scale, the respondents classified cancer as a major contributor to disease burden (median 10, IQR 9–10). Cardiovascular disease (CVD) was ranked second (median 9, IQR 8–10), and TBDs—third (median 8, IQR 6–9) (Figure 2). The differences were statistically significant with *p* < 0.001 in one-way mixed-effects ANOVA and Tukey’s multiple comparisons post hoc test. Interestingly, TBDs were rated equal to or higher than cancer and CVD by 39% and 46% of the respondents, respectively. Overall, 50% of the respondents rated TBDs as equal to or more significant than cancer or CVD. Respondents rating TBDs at least equally high to cancer or CVD did not differ in terms of age, sex, place of residence, exposure to ticks, tick bite history, and GKS scores. There was a difference in education levels. Having a university diploma was associated with perception of a lower disease burden attributed to TBDs (42.6% with university diploma and 62.6% without it ranked TBDs as at least equal to cancer and CVD; chi-square statistic 13.96; *p* < 0.001).

After a tick bite, 50% of surveyed individuals remove the tick by themselves, 47% wanted to have the tick removed by a healthcare professional, 2% want to do both, and only 1% want to deter the tick by covering it with oil or butter. A total of 69% answered that they would like to test the removed tick for pathogens. In addition, a total of 88.3% of the respondents were convinced that mouthparts remaining in the skin after tick removal pose a significant health danger (7 points or more in the 10-point Likert scale). The median result was 10 (IQR 8–10). Surveyed individuals who attributed lower risk to tick fragments in the skin were also more likely to remove a tick by themselves, without consultation with a healthcare professional (OR, 2.67; 95% CI, 1.19–5.97; *p* = 0.018). Only 14.7% of respondents believe that post-exposure prophylaxis with antibiotics should always be administered after a tick bite, and 33.8% admit that they “do not know”. Post-exposure prophylaxis with an antibiotic was not commonly used after tick bites. Out of those bitten by a tick in the past, only 19 (12%) respondents and 10 (8%) of respondents’ children received a prophylaxis with an antibiotic after a tick bite.

We also asked the respondents to assess their own knowledge about TBDs and the principles of their prevention. As assessed with the 10-point Likert score, respondents feel moderately knowledgeable with regard to general knowledge about TBDs (median 5, IQR 4–6) and knowledge about tick bite prophylaxis (median 6, IQR 5–8). Thirty-six percent of the respondents feel confident about the prophylaxis and TBDs (score 7 or higher). These respondents tended to score higher results in GKS (OR, 2.5; 95% CI 1.5–4.1, *p* < 0.001) and also tended to assign higher disease burden scores to TBD, in relation to CVD and cancer (OR 1.7, 95% CI 1.1–2.8, *p* = 0.02).

### 3.4. Practice

The median TPS was 63.6% (IQR, 54.5%; 72.7%). Respondents with TPS below 25th percentile (50% points or less out of 11) were categorized as having poor knowledge of tick bite protection. Low TPS received 18.7% of the respondents. In the multivariate model, poor tick bite protection was associated with the male sex (OR 2.36, 95% CI 1.10–5.09, *p* = 0.028) and lower education levels (OR, 2.48; 95% CI, 1.30–4.73; *p* = 0.006). On the contrary, respondents whose child was bitten by a tick were less likely to score low TPS (OR 0.21, 95% CI 0.09–0.50, *p* < 0.001). Place of residence, and spending time in outdoor green spaces were not associated with tick protection practices. 

Surveyed individuals with high TPS scored high GKS more often, indicating a connection between knowledge and good preventive practices in a univariate model (OR, 1.90; 95% CI, 1.12–3.25; *p* = 0.02). However, when the history of tick bites, exposure to ticks in green areas and other confounders were included in the multivariate model, high GKS was no longer a predictor of proper preventive practices expressed as high TPS. 

**Figure 3:**
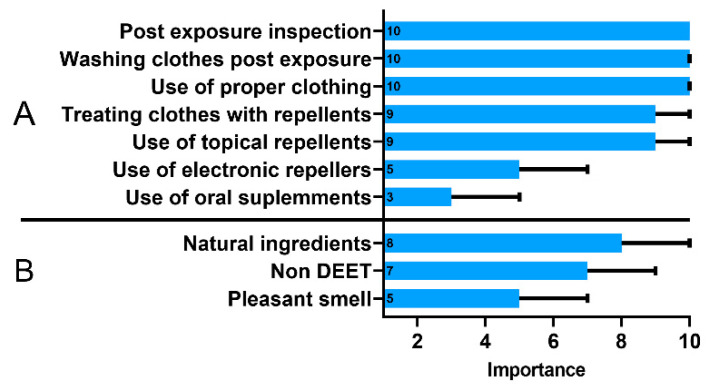
The importance of tick protection methods (**A**) and characteristics of a tick repellent (**B**) assessed using the 10-point Likert scale ranging from 1—“of little importance” to 10—“highly important”. Data presented as medians (boxes) and interquartile range (whiskers). The annotations within the bars represent the median values.

The respondents recognized that post-exposure inspection of the body after returning from green areas and using appropriate clothing and washing clothes were more important methods of prophylaxis than the use of repellents on the body or clothing (Figure 3A). The use of oral supplements and electronic repellants, which are considered substantially less effective in tick protection compared to the other methods, was not preferred by the respondents. Only 61% (248/406) of the respondents purchased a repellent in the last year, and when choosing a product, 77% (304/394) of them were persuaded by its natural composition (Figure 3B). Based on these responses, we calculated an additional index regarding use of repellents. As mentioned before, individuals with high GKS were less likely to have a poor repellent practice (OR, 0.58; 95% CI, 0.36–0.94).

The attitude of the respondents to TBE vaccination was also assessed. A positive attitude towards TBD vaccine (modeled as receiving a TBE vaccine in the past, vaccinating a child with the TBE vaccine in the past, or an intention to receive this vaccine or to vaccinate a respondent’s child in the future) was observed in 48.0% (191/398) and was more common in respondents with a recent tick bite (OR 2.10, 1.07–4.10, *p* = 0.030), in respondents whose child was bitten by a tick (OR, 1.69; 95% CI, 1.00–2.83, *p* = 0.049), and in respondents who relied on verified sources of information (OR, 2.654, 1.66–4.23; *p* < 0.001). 

## 4. Discussion

Our findings demonstrated that inhabitants of northeastern Poland, where TBDs are endemic have moderate knowledge about TBDs, including symptoms, transmission, treatment and prevention. Contrary to other studies [29,30,31], calculated scores were not particularly low in our study, despite the fact that we surveyed general population, and not professionals working in green areas, who are usually better informed about TBDs. However, we identified multiple gaps in knowledge and misconceptions regarding TBDs, which might pose a real health risk.

Almost a quarter of the surveyed individuals incorrectly indicated a tick in an illustration. Additionally, the majority of respondents believed that leaving a tick fragment in the skin is associated with a high health risk, which in turn was associated with the desire to have the tick removed by a healthcare professional. Seeking medical advice, whether in emergency care or general practice, extends the time the ticks remain in the skin, which increases the risk of LB infection [32].

Alongside antibiotics, almost half of the study participants chose other non-verified treatment options for LB. This possibly reflects a false belief, that LB patients often experience many persistent symptoms after infection with *Borrelia burgdorferi*, and as a result require additional treatment. Accordingly, a minority of respondents in this study were convinced that LB is a curable disease. It is not surprising then that the respondents perceived TBDs as almost as dangerous as CVD and cancer. It was shown previously that patients with chronic LB have significantly impaired quality of life and greater healthcare utilization compared to the general population and patients with other chronic diseases [33]. However, persistent symptoms are reported in a relatively small subset of treated LB patients [32].

The increasing problem with ticks and LB triggered efforts to develop vaccines protecting from infections with *Borrelia burgdorferi*. Currently, while awaiting a widely accepted vaccine against LB, the only method of protection against LB is avoiding tick bites through the use of appropriate personal protection measures [34,35]. There is no single best measure to protect from tick bites. A combination of two or more actions, such as wearing light-colored clothing that cover legs and arms, changing clothes worn outdoors, checking body for ticks and removing them promptly, and applying tick repellents on skin and clothes provides the best protection against tick bites [36]. Not surprisingly, good tick protection practices were more common in individuals whose child was bitten by a tick. Increased risk perceptions are associated with adoption of behaviors reducing tick bites, what was demonstrated in multiple previous studies [36]. In this study the use of tick repellents on the body or clothing was considered less important than other personal protection measures to prevent tick bites. Similar results were obtained in the Netherlands and the USA. These studies showed that respondents preferred wearing appropriate clothing, body checking and prompt tick removing over the use repellants on clothing or skin [28,29,37]. In addition, our study showed that although the respondents live in an endemic area with high tick activity, only 61% of them bought a repellent in the last year, and when choosing a product, 77% were persuaded by its natural composition and lack of N, N-diethyl-m-toluamide (DEET). Research has shown that respondents are reluctant to use repellants, which may be related to the commonly known side effects of using DEET [38]. Respondents mistakenly choose natural repellants, not knowing that their deterrent effect is uncertain. Moreover, such a choice may be dictated by the ignorance of the proper use of DEET preparations, as discussed by the Center for Disease Control and Prevention (CDC) and the Environmental Protection Agency, with detailed recommendations for use in children [38]. 

Prophylaxis with a single or multiple doses of doxycycline to prevent Lyme disease after a tick bite remains controversial. Using antibiotic prophylaxis might be advantageous, but further confirmation is needed [39]. According to the guidelines, post-exposure prophylaxis should be considered in individual cases, taking into account multiple tick bites during a stay in an LB endemic area [40].

Respondents of our study rarely receive antibiotics after a tick bite and are not convinced that antibiotic prophylaxis should always be used after being bitten by a tick. They commonly, however, wanted to seek medical advice either to have the tick removed from the skin or to test it for infections. Therefore, health care professionals play an important role in communicating proper tick bite management. In the previous study conducted in the USA, assessing the knowledge and challenges in the diagnosis and treatment of TBDs among doctors, it was found that emergency care and emergency medicine specialists prescribe treatment incompatible with the guidelines for the prevention of tick bites [41], and healthcare providers from areas with low incidence of LB in the USA too often use post-exposure prophylaxis in the form of antibiotic therapy [42]. Moreover, clinicians from the United States complained about difficulties in communicating with patients, resulting from the patients’ misconceptions derived from sources other than guideline-based resources and evidence [41]. 

Our study showed that the acceptance of the TBE vaccine is low in general population. A positive attitude towards this vaccine was more common in respondents with a recent tick bite, what may be associated with a higher risk perception of TBDs. Other studies also show that people at risk of the disease most often express the willingness to be vaccinated [43,44]. Therefore, communicating risks of LB might increase the public acceptance of the future LB vaccine. In one study assessing tick-related KAP among people living in the endemic areas of Connecticut and Maryland, the respondents were asked about preventive behaviors for LB, including a desire to receive a vaccine against LB if one was available. Eighty- four percent of the respondents declared that they were moderately (35%) or very (49%) willing to accept the LB vaccine, but they claimed that body checking and bathing after coming from green areas is more important [26]. A recent survey conducted in the USA showed that willingness to receive a Lyme disease vaccine was high [45]. The results of our research, as well as the data from the literature, suggest that as the work on the development of vaccines progresses, further research is needed to determine the causes of negative attitudes towards vaccination in part of the population living in endemic areas. This will allow for the preparation of optimal educational activities promoting vaccination against both TBE and LB, if a vaccine for the latter is registered and approved for use.

Previous studies on TBD knowledge, attitude and practice have produced mixed results. Some of them showed that the society is well educated in the field of TBD threats [46], while others, just like in our study, showed that the knowledge in this area is insufficient [30,47]. We should acknowledge several limitations of our study. Firstly, it should be noted that women are overrepresented in this study, thus our results should not be generalized to the entire population. Secondly, correlations we calculated do not necessarily mean causation, as there might be other undiscovered factors that have an impact on preventive behaviors and attitudes. However, knowledge gaps we identified and associations we found might be addressed in future educational campaigns. Previously it was shown that recognizing the gaps in knowledge may be helpful in planning an appropriate educational program, raising awareness of LB and tick bite prevention among the general population. A study conducted in France in 2016–2019 showed that the implementation of a national plan against tick-borne infections resulted in a greater proportion of the population applying protective measures against tick bites and tick-borne diseases [24].

## 5. Conclusions

The respondents living in the endemic region of northeastern Poland with high tick activity consider TBDs a significant threat to the health of themselves and their families. People who have been bitten by a tick personally or whose children have experienced it know the most about TBDs. Gaps in knowledge and misconceptions regarding the prevention of LB require the implementation of educational activities. Additionally, understanding gaps in knowledge and practice will allow for proper planning of educational activities, which could contribute to inhibiting the increase in the incidence of LB, in anticipation of an effective and socially acceptable vaccine. 

Modification of educational activities should take into account the use of the mass media and the Internet, which are currently the main information channels for the respondents. The information campaign should also include facts about TBD immunoprophylaxis with both the registered TBE vaccine and the future LB vaccine.

## Figures and Tables

**Table 1:** Personal characteristics of the respondents.

Individual—Related Characteristic	Number (%)
**Gender** Male Female	**406 (100%)**
	72/397 (18%)
	325/397 (82%)
**Age** <29 30–39 40–49 >49	**400 (100%)** 74/400 (18%) 235/400 (59%) 79/400 (20%)
	12/400 (3%)
**Education** Primary Secondary Vocational Higher	**400 (100%)** 10/400 (2%) 88/400 (22%)
	37/400 (9%)
	267/400 (67%)
**Place of residence** **Rural** **Urban**	**397 (100%)** 165/397 (42%) 232/397 (58%)
**Bitten by a tick in the past**	161/388 (41%)
**Bitten by a tick in the last year** **at least once**	66/402 (16%)
**Bitten by a tick in the last year** **more than once**	21/405 (5%)
**Respondent’s child bitten by a tick** **in the past**	123/393 (31%)
**Respondent’s child bitten by a tick** **more than once**	40/405 (10%)

Data presented as frequencies and percentages. Denominators lower than the study group indicate missing data.

## Data Availability

The datasets used and/or analyzed during the current study are available from the corresponding author on reasonable request.
